# Vitamin D3 for the Treatment of Epilepsy: Basic Mechanisms, Animal Models, and Clinical Trials

**DOI:** 10.3389/fneur.2016.00218

**Published:** 2016-12-08

**Authors:** Kevin Pendo, Christopher M. DeGiorgio

**Affiliations:** ^1^Princeton University, Princeton, NJ, USA; ^2^Department of Neurology, University of California Los Angeles, Los Angeles, CA, USA

**Keywords:** cholecalciferol, vitamin D3, epilepsy, SUDEP, animal models, clinical trials

## Abstract

There is increasing evidence supporting dietary and alternative therapies for epilepsy, including the ketogenic diet, modified Atkins diet, and omega-3 fatty acids. Vitamin D3 is actively under investigation as a potential intervention for epilepsy. Vitamin D3 is fat-soluble steroid, which shows promise in animal models of epilepsy. Basic research has shed light on the possible mechanisms by which Vitamin D3 may reduce seizures, and animal data support the efficacy of Vitamin D3 in rat and mouse models of epilepsy. Very little clinical data exist to support the treatment of human epilepsy with Vitamin D3, but positive findings from preliminary clinical trials warrant larger Phase I and II clinical trials in order to more rigorously determine the potential therapeutic value of Vitamin D3 as a treatment for human epilepsy.

## Introduction

Epilepsy affects approximately two million Americans and 65 million people worldwide (1). Among those with epilepsy, 22–30% have drug-resistant epilepsy (DRE) (1, 2). DRE causes cognitive and mood impairment, injuries, and increased risk of death including sudden death in epilepsy (SUDEP) (1–3). Antiepileptic drugs (AEDs) are the primary medical treatment for epilepsy. However, even for those whose seizures are well controlled by AEDs, allergies, neurological and systemic toxicity, depression, memory loss, and osteoporosis are common problems (4, 5). Because of the limitations and potential toxicity of existing AEDs, there is significant clinical interest in finding alternative therapies for epilepsy.

In the search for alternative epilepsy treatments, Vitamin D3 is an intriguing candidate (6). As early as 1974, Christiansen postulated that supplementation of Vitamin D might improve calcium and magnesium levels and may decrease hyperexcitability in patients with epilepsy. In the four decades since, progress has been made in understanding the biochemical and cellular mechanisms of Vitamin D3’s anticonvulsant properties. Animal data have supported the anticonvulsant effects of Vitamin D3 in mice and rats (7–11). Existing evidence for the use of Vitamin D3 in treating human epilepsy is very limited (6, 12). There is a critical need for larger clinical trials to establish the safety and efficacy of vitamin D3 in epilepsy. In this review, we will critically analyze the animal and human evidence to date supporting the use of Vitamin D3 as a treatment for epilepsy.

## Vitamin D3 Overview: Biochemistry and Role in Human Health

**Figure 1:**
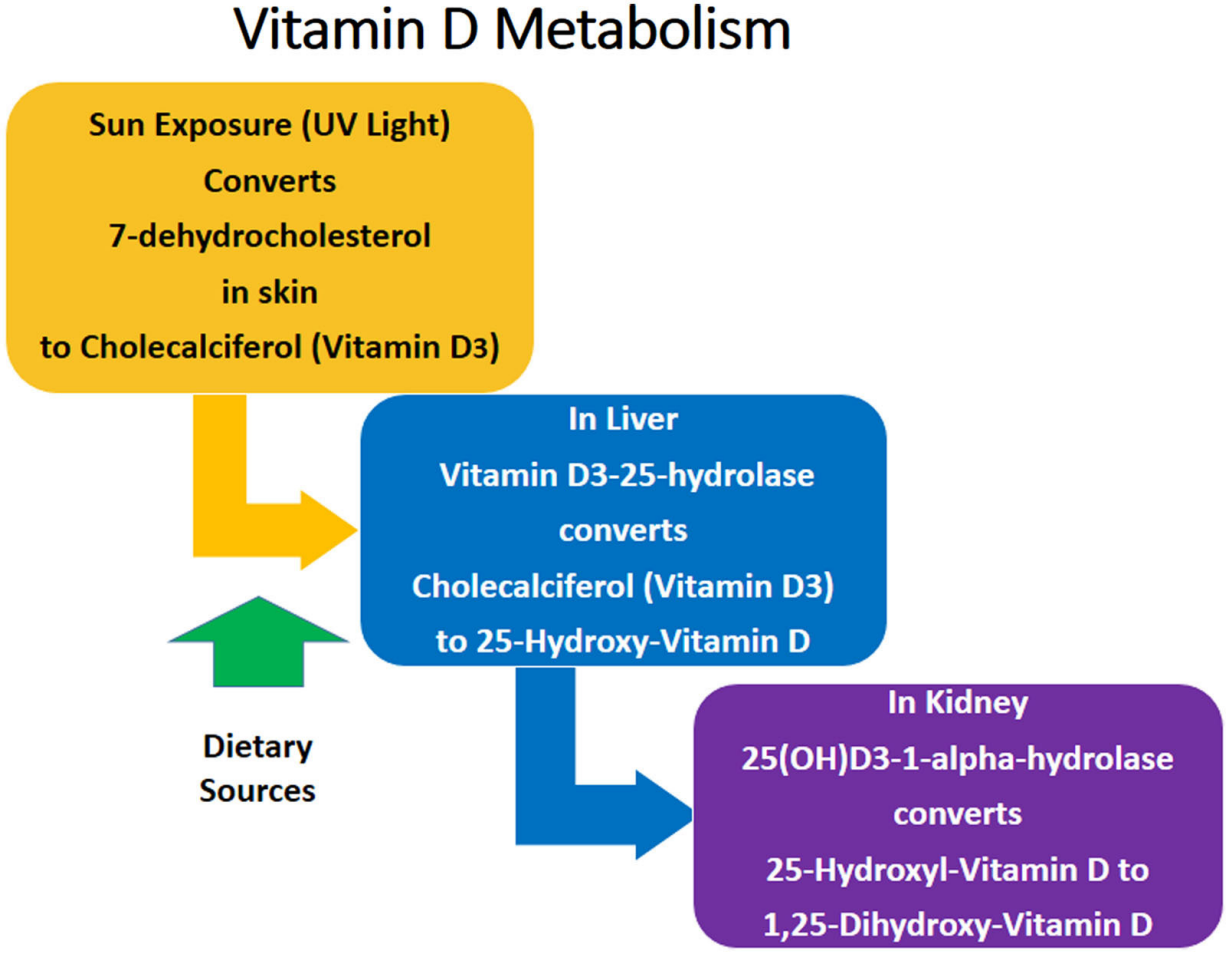
**Vitamin D metabolism**.

The most biologically active form of Vitamin D in humans is Vitamin D3 (cholecalciferol), which is a fat-soluble steroid hormone (13). Dietary sources of Vitamin D3 include dairy, meat, fish, and mushrooms (14). The primary source of Vitamin D3 is exposure of the skin to ultraviolet sunlight (14). The metabolic pathway of Vitamin D3 is summarized in Figure 1. 7-dehydrocholesterol is converted to Vitamin D3 in the skin after exposure to sunlight. Vitamin D3 is converted to 25-hydroxy-cholecalciferol (25-OH Vitamin D3) in the liver. 25-OH Vitamin D3 is the major circulating form of Vitamin D, but it itself is biologically inactive and must be converted to the active form 1,25-dihydroxy-Vitamin D3 (1,25 Vitamin D3) in the kidneys (13–15). Vitamin D3 is important for calcium metabolism, bone health, cardiac function, and blood pressure maintenance, among other health benefits (14, 16, 17). Vitamin D3 deficiency is a marker of poor health and overall mortality (16). However, 40–50% of Americans have insufficient Vitamin D3 levels, and insufficiency is even more prevalent in underserved populations, including Hispanics (69%) and African Americans (82%) (18).

## Vitamin D3 in the Brain and Nervous System

Among its variety of health benefits, Vitamin D3 plays an important role in the human brain and nervous system, as indicated by increasing evidence gathered over the past several decades. Researchers have explored the role of Vitamin D3 in Alzheimer’s disease and dementias (19, 20), Parkinson’s disease (19, 21), multiple sclerosis (22–24), schizophrenia (25), affective disorders (13, 26), cognitive decline (13, 27), and epilepsy (6, 12). Vitamin D3 is also involved in neuroprotection (15, 28, 29), brain cell proliferation and differentiation (30, 31), and brain development (30, 32, 33). A neurological role of Vitamin D3 is further supported by the presence of Vitamin D3-specific receptors and enzymes in neurons and glial cells throughout the brain, in the spinal cord, and in the peripheral nervous system (34–37). The broad role of Vitamin D3 in the nervous system has engendered research into Vitamin D3’s anticonvulsant action in the brain, and the proposed mechanisms of action can generally be categorized as either genomic or non-genomic.

### Genomic Mechanisms of Action

Genomic mechanisms behind Vitamin D3’s anticonvulsant effect are based on Vitamin D3’s ability to regulate the expression of genes, a process that is mediated by a nuclear Vitamin D3 receptor (VDR) (38). VDR is a ligand-specific transcription factor, which is activated by Vitamin D3 and subsequently alters gene expression (28, 29). Through this mechanism, Vitamin D3 lowers the expression of certain proconvulsant cytokines, such as IL-1β and TNF-α. These cytokines can increase seizure susceptibility in several ways. IL-1β is involved in a pathway that results in phosphorylation of the NR2B subunit of the NMDA receptor, which is a glutamate receptor that is important in the generation of seizures (39). The phosphorylation of this NMDA receptor subunit increases Ca^2+^ influx into neurons (40) and stabilizes the receptor in the membrane (41), leading to the hyperexcitability of neurons that can cause seizures (39, 42). IL-1β can also cause neuronal hyperexcitability by increasing the release probability of glutamate (43), an excitatory neurotransmitter, and inhibiting its reuptake (39, 44). In addition, IL-1β can reduce inhibitory GABA-ergic Cl^−^ flux (45), furthering the proconvulsant effect of this cytokine (39). The TNF-α cytokine acts as a proconvulsant because it initiates both the recruitment of AMPA receptors to the neuronal membrane and the endocytosis of GABA_A_ receptors away from the membrane (46, 47). The TNF-α-induced overexpression of AMPA receptors and under-expression of GABA_A_ receptors on the neuronal membranes results in more excitatory synaptic transmission and less inhibitory signaling, which increases the likelihood of epileptic activity.

Through its nuclear VDR, Vitamin D3 can also increase the expression of anticonvulsant growth factors GDNF and NT3 (15, 29, 48–50). NT3 leads to an anticonvulsant effect by downregulating TrkA and TrkC receptors, which are receptors that regulate synaptic strength (50). The mechanism behind GDNF’s anticonvulsant action remains largely unknown, but it is speculated that, similar to that of NT3, it involves some modulation of synaptic transmission (51). Vitamin D3-activated VDR also promotes expression of the calcium-binding proteins parvalbumin and calbindins, which inhibit epileptic episodes (15, 29, 52). By binding to Ca^2+^ in the presynaptic terminal, these calcium-binding proteins prevent excessive Ca^2+^-induced neurotransmitter release and thus protect against epileptic activity (52, 53).

### Non-Genomic Mechanisms of Action

Faster, non-genomic mechanisms of Vitamin D3’s anticonvulsant effect have been proposed as well. Vitamin D3’s ability to increase calcium uptake from the intestine can alter plasma and brain Ca^2+^ concentrations, which may decrease neuronal excitability and prevent seizures. However, evidence suggests that Vitamin D3’s anticonvulsant effect is not wholly attributable to its role in altering calcium levels (6, 8, 9). Rather, it is more likely that Vitamin D3’s rapid, anticonvulsant effect results from its ability to fine-tune Ca^2+^ and Cl^−^ currents across neuronal membranes (54, 55). Vitamin D3 initiates non-genomic signal transduction pathways that ultimately alter the conductance of L-type calcium channels and chloride channels, therefore affecting neuronal excitability and seizure susceptibility at the threshold level (55–57). The details of these non-genomic signal transduction pathways are debated, and although it used to be thought that they were mediated by a distinct membrane Vitamin D3 receptor (VDR_mem_) (58), more recent evidence suggests that these rapid, non-genomic anticonvulsant pathways are actually mediated by the same protein – VDR – that mediates Vitamin D3’s genomic actions (54, 57, 59–61), with different domains of VDR being involved in the genomic and non-genomic pathways that lead to Vitamin D3’s anticonvulsant effects.

## Vitamin D3 in Animal Models of Seizures

### Rat Models

In 1984, Siegel et al. published a seminal paper describing the effect of Vitamin D3 on seizure thresholds in rat hippocampi (7). Using artificial electrical stimulation to model seizures, they found that stereotactic injection of 50 or 100 μg of 1,25 Vitamin D3 into the hippocampus of rats significantly elevated the seizure threshold in all rats treated. This elevation in threshold was noticeable 5–10 min after the injection of 1,25 Vitamin D3, and the effect lasted at least 120–180 min. Intravenous injection of 1,25 Vitamin D3 also significantly elevated seizure threshold, but the effect was transient, lasting only 30 min, perhaps due to limited uptake of 1,25 Vitamin D3 in the brain. Most rats were Vitamin D3-sufficient, but they found that in one Vitamin D3-deficient rat, a lower dose of 1,25 Vitamin D3 was required to raise the seizure threshold to a similar extent.

### Mouse Models

Over two decades after Siegel et al.’s rat study, Kalueff et al. explored the anticonvulsant effects of Vitamin D3 in a mouse model of seizures (8). Subcutaneous injection of 33 μg of 1,25 Vitamin D3 incurred an anticonvulsant effect in a chemically induced model of seizures. Compared to controls, mice injected with 1,25 Vitamin D3 40 min prior to the injection of pentylenetetrazol (PTZ), a seizure-inducing chemical, exhibited longer mean latency to seizure onset (77 vs. 55 s), shorter mean duration of tonic–clonic seizures (10 vs. 32 s), and lower mortality (18 vs. 55%). However, the anticonvulsant effects of 1,25 Vitamin D3 were nearly gone if Vitamin D3 injection occurred 3, 6, 12, or 24 h before PTZ injection. The acute efficacy of 1,25 Vitamin D3 suggests that the anticonvulsant effect in this model was due to non-genomic actions of the steroid. In addition, differences in Ca^2+^ levels between control and experimental mice were non-significant, suggesting that 1,25 Vitamin D3 exerted an anticonvulsant effect independent of its role in calcium metabolism (8).

In a separate study, Kalueff et al. found that the partial deletion of the VDR gene in mice led to increased seizure severity in the model of PTZ-induced seizures (9). Compared to wild-type mice, VDR-knockout mice demonstrated significantly shorter latencies to seizure onset (50.4 vs. 66.9 s), higher Racine scores of seizure severity (5.9 vs. 4.9), and increased mortality (90 vs. 40%). Of note, none of the mice in either the control or experimental condition showed spontaneous seizure activity, suggesting that the VDR gene acts at the threshold level of seizures. Both wild-type and VDR-knockout mice had normal calcium levels, suggesting that the partial deletion of the VDR gene increases seizure intensity *via* a non-calcium mechanism and providing further evidence of an anticonvulsant effect of Vitamin D3 that is independent from its role in calcium metabolism (9).

In two studies, Borowicz et al. have shown that certain doses of Vitamin D3 enhance the efficacy of several AEDs in a mouse electroshock model of epilepsy without altering the concentrations of the drugs, suggesting a synergistic pharmacological interaction (10, 11). The authors also reported some anticonvulsant action of Vitamin D3 in its own right (10), and they found that treatment with Vitamin D3 led to no deleterious changes in motor coordination, long-term memory, or anxiety (10, 11).

Overall, existing evidence from rat and mouse studies supports an acute anticonvulsant effect of Vitamin D3 in electric shock and chemically induced models of seizure. Further research is needed to explore the longer-term effects of Vitamin D3 therapy in diverse animal models of epilepsy.

## Vitamin D3 in Human Epilepsy

People with epilepsy are often Vitamin D3-deficient, along with having decreased bone density and higher rates of osteoporosis (62). Furthermore, certain AEDs, such as carbamazepine and phenytoin, are known to decrease Vitamin D3 levels in people who are taking them due to increased metabolic clearance of Vitamin D3 and conversion to inactive forms (63, 64). People with epilepsy face a sixfold risk for bone fracture compared to the normal population, likely an interplay between frequent falls, reduced bone density, and low levels of Vitamin D3 (62). Maternal Vitamin D3 deficiency during pregnancy has also been associated with hypocalcemia-induced seizures in neonates, which have been successfully treated with calcium and Vitamin D3 supplementation in several case studies (65–68).

In humans, little clinical data exist about the effect of Vitamin D3 supplementation on seizures. In 1974, Christiansen et al. conducted a pilot study in which they treated 23 epilepsy patients with Vitamin D3 (6). Subjects were divided into two groups (A and B) for the duration of the 12-week study, which was divided into a 4-week observation phase (T1) followed by two 4-week treatment periods (T2 and T3). Group A (*n* = 9) received 4,000 IU/day of Vitamin D3 during T2, followed by 16,000 IU/day during T3. Group B (*n* = 14) received placebo during T2, followed by 8,000 IU/day of Vitamin D3 during T3. During T2, Group A (treated with 4,000 IU/day of Vitamin D3) experienced a mean reduction in seizure frequency of 32% from baseline, while Group B (placebo) experienced an 8% reduction in mean seizure frequency from baseline. During T3, Group A (treated with 16,000 IU/day of Vitamin D3) experienced a 29% reduction in mean seizure frequency from baseline, while Group B (being treated with 8,000 IU/day of Vitamin D3) experienced a similar. In both groups, high dose vitamin D3 (8000 to 16000 IU/day) was associated with reductions in seizure frequency 33% reduction in mean seizure frequency from baseline. The authors concluded that high dose Vitamin D3 significantly reduced the number of seizures in patients with poorly controlled epilepsy, and, contrary to the authors’ hypothesis, it did so independently of calcium or magnesium levels (6).

Nearly 40 years after Christiansen et al.’s findings, Holló et al. conducted the most recent clinical study of Vitamin D3 therapy in human epilepsy (12). Their subjects consisted of 13 patients with DRE. At baseline, low 25-OH-Vitamin D3 levels <30 ng/ml were present in 12/13 patients and deficient levels (<12 ng/ml) were present in 8/13 patients; 1/13 patients had a normal Vitamin D3 level at baseline. Treatment consisted of Vitamin D3 supplementation aimed at normalizing the serum Vitamin D3 levels of all the subjects. To the 12 patients with low or deficient Vitamin D3 levels at baseline, an oral dose of 40,000–200,000 IU bolus of Vitamin D3 was administered, and treatment was continued with a daily maintenance dose of 2,000–2,600 IU/day of Vitamin D3. The one subject with normal baseline Vitamin D3 level only received the daily maintenance doses. Vitamin D3 levels were rechecked 3 months after treatment onset to determine successful normalization of Vitamin D3 levels and rule out potential Vitamin D3 toxicity. Vitamin D3 supplementation was determined to be safe, as no subjects showed toxic levels of Vitamin D3 at the 3-month follow-up (12). Median Vitamin D3 level rose from 11.8 ng/ml at baseline (range: <4–34.2 ng/ml) to 38.0 ng/ml at 3-month follow-up (range: 23.3–45.0 ng/ml). This elevation in Vitamin D3 levels was significant (*p* = 0.001, sign test), and the posttreatment Vitamin D3 levels of all subjects were within or close to the normal range (12). The efficacy of the Vitamin D3 treatment in reducing seizures was determined by comparing the number of seizures experienced during the 90 days prior to treatment onset to the number of seizures experienced in the 90 days after treatment onset. Among all subjects, 10/13 experienced fewer seizures after initialization of Vitamin D3 treatment, 2/13 had more seizures, and 1/13 had the same number of seizures. The median reduction in seizure number following treatment onset was 40% and was significant (*p* = 0.04). In addition, 5/13 patients experienced a ≥50% reduction in number of seizures. The existing clinical evidence suggests a therapeutic effect of Vitamin D3 in human epilepsy, but there is a need for larger Phase I trials and Phase II randomized, placebo-controlled trials to investigate optimal dosing and short-term and long-term efficacy.

## Does Vitamin D3 have a Potential Role in Reducing Sudep Risk?

Vitamin D3 status is strongly associated with risk of sudden cardiac death in heart disease and patients with severe kidney disease on hemodialysis. In a large prospective study of 2,300 patients in the Cardiovascular Health Study, the risk of sudden cardiac death was 2-times higher in those with Vitamin D3 levels <20 ng/ml (4 events/1,000) than in those with Vitamin D3 levels >20 ng/ml (2 events/1,000) (69). Similarly, in a study of 1,108 patients with chronic kidney disease, very low levels of Vitamin D3 (25-hydroxy-Vitamin D3 levels <25 nmol/l) were 3-times more likely to sustain sudden cardiac death than those with high levels >75 nmol/l (hazard ratio = 2.99) (70).

Common to severe heart and kidney disease is impaired heart rate variability (HRV), particularly vagus-mediated high-frequency HRV (69–72). Patients with DRE, who are at high risk for SUDEP, have impaired vagus-mediated HRV, similar in magnitude to patients with heart failure (69, 70, 73, 74). Recently, subjects with DRE, at high risk of SUDEP, as measured by the SUDEP-7 inventory, were found to have severe impairment in RMSSD, a measure of vagus-mediated HRV (73, 74). In a recent study linking SUDEP risk in patients with DRE, those with the highest SUDEP-7 inventory risk scores in the highest quartile had RMSSD values of 17.6 ms, vs. 32.0 ms for those with the lowest SUDEP-7 inventory scores (*p* = 0.03, trend test) (74). This finding is relevant since Vitamin D3 supplementation improves vagus-mediated HRV (71, 72, 75). Recently, Vitamin D3 supplementation ranging from 5,000 to 10,000 IUs in normal controls resulted in significant improvements in high-frequency HRV, as measured by the low-frequency/high-frequency HRV ratio (75). A similar result was recently found in patients with IGA nephropathy, where high-frequency HRV, as measured by the LF/HF HRV ratio, also increased after Vitamin D3 supplementation (71).

## Conclusion and Future Directions

The weight of evidence from basic research and animal models over the past several decades supports an anticonvulsant effect of Vitamin D3. Vitamin D3’s anticonvulsant action may be *via* genomic and non-genomic mechanisms. Epidemiological data as well as a variety of case studies also point to a connection between Vitamin D3 and epilepsy and support the use of Vitamin D3 as a potential therapy for human epilepsy, both in its own right and in conjunction with existing AEDs. However, the clinical data that exist are limited by small sample size and/or lack of randomization and double-blind placebo control. Despite these limitations, existing clinical data have, in the opinion of this review, been positive enough to warrant larger Phase I and Phase II clinical trials in order to more rigorously determine the potential therapeutic value of Vitamin D3 as a treatment for human epilepsy. Recently, our group has received an IND for a Phase I study of Vitamin D3 in DRE to study the safety, preliminary efficacy, and potential cardiac benefits of Vitamin D3 5,000 IU/day in DRE.

## Author Contributions

The authors have contributed to the preparation, research, and writing of the manuscript.

## Conflict of Interest Statement

The authors declare that the research was conducted in the absence of any commercial or financial relationships that could be construed as a potential conflict of interest.
